# Clinical, genetic, radiological characteristics and management of mediastinal paragangliomas: a literature review and case series

**DOI:** 10.1530/ERC-24-0279

**Published:** 2025-03-24

**Authors:** Mark Quinn, Yasmine Kemkem, Gemma White, Phil Touska, Dimitra Christodoulou, Audrey Jacques, Louise Breen, Barbara McGowan, Mamta Joshi, Fahim Ul Hassan, Karen Harrison-Phipps, Johnathan G Hubbard, Rupert Obholzer, Louise Izatt, Paul Carroll, Anand Velusamy

**Affiliations:** Departments of Endocrinology, Radiology, Nuclear Medicine, Cardio-Thoracic Surgery, Genetics, Endocrine Surgery and ENT, Guy’s and St Thomas’ NHS Foundation Trust, London, UK

**Keywords:** paraganglioma, mediastinal, SDHB, SDHD, neuroendocrine tumours

## Abstract

Paragangliomas (PGLs) are neuroendocrine tumours (NETs) that arise from neural crest-derived cells. Up to 40% of cases occur due to the presence of a pathogenic germline variant (PGV) in a known gene. Mediastinal PGLs are rare but are being diagnosed with increasing frequency. Treatment generally involves surgery but is complicated in mediastinal PGLs due to their anatomy. Here, we will perform a literature review and discuss our experience with 18 such cases. Cases were identified via the Guy’s and St Thomas’ NHS Foundation Trust NET multidisciplinary team database. Tumours ranged in size from 0.6 × 0.6 to 6.8 × 4.9 cm. 72.2% were associated with a PGV of *SDHB* or *SDHD*. 22.2% developed metastatic disease, but it was only possible to attribute 50% of these to a mediastinal primary. ^68^Ga-DOTATATE PET CT demonstrated 100% sensitivity. The literature review identified 233 cases. A PGV was reported in 81% of cases, with metastatic disease in approximately 39.2%. It was not possible to confirm that all cases of metastatic disease were secondary to a mediastinal primary. Our experience confirms the high rate of mediastinal PGLs arising in the presence of a PGV. The lower rate of metastatic disease in our cohort (11.1%) likely represents earlier diagnosis thanks to the application of screening protocols and the increased sensitivity of ^68^Ga-DOTATATE PET CT. With this increased sensitivity, we have diagnosed small mediastinal PGLs that were not evident on alternative imaging modalities. In the absence of growth or catecholamine secretion, the need to intervene on these is unclear.

## Introduction

**Figure 1:**
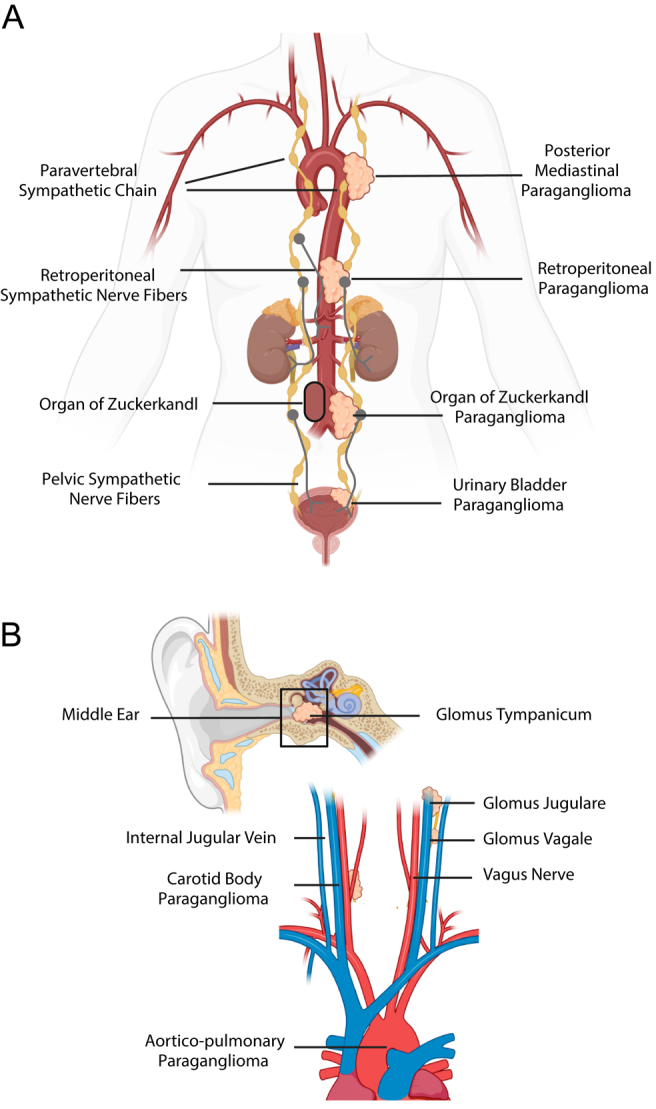
Sympathetic and parasympathetic paraganglioma locations within the (A) thorax, abdomen and pelvis, and (B) head and neck. Created in https://BioRender.com.

Paragangliomas (PGLs) are rare neuroendocrine tumours that arise from the autonomic paraganglia and can occur anywhere these cells exist, from the base of the skull to the pelvis (Fig. 1). When they occur in the adrenal medulla, they are called phaeochromocytomas (PCCs). Collectively, these tumours are referred to as PPGLs. PPGLs are among the most inherited of all tumour subtypes, with at least 40% occurring due to the presence of a pathogenic germline variant (PGV) in a known susceptibility gene (Fishbein *et al.* 2013). Many of these PGVs have been discovered in recent years and are most often associated with an autosomal dominant inheritance pattern. With each PPGL index case, there are often numerous family members identified as PGV carriers via cascade screening. The penetrance of each PGV is variable (Table 1), and we currently have no way of predicting which patients are at risk of developing the disease. Current guidelines therefore dictate that all PGV carriers undergo a full-body MRI at diagnosis and then every 2–3 years for the duration of their follow-up, which is likely to be lifelong (Amar *et al.* 2021).

**Table 1:** The current list of PGVs known to be associated with PPGLs, their status on the current NHS panel, their inheritance pattern and their estimated penetrance where known.

PGV	Year discovered	Included on NHS panel (R223)	Mode of inheritance	Approximate penetrance (relating to PPGLs)
*DLST*	2019	Yes (2021)	Autosomal dominant (AD)	Unknown (predicted low)
*FH*	2014	Yes (2019)	AD	Unknown
*MAX*	2011	Yes (2019)	AD	Unknown
*MDH2*	2018	Yes (2021)	AD	Unknown
*MEN1*	1997	Yes (2019)	AD	<2% (Machens *et al.* 2007)
*RET*	1985	Yes (2019)	AD	32% (Quayle *et al.* 2007)
*SDHA*	2010	Yes (2019)	AD	<5% (Maniam *et al.* 2018)
*SDHAF2*	2010	Yes (2019)	AD. Possible paternal transmission of tumour susceptibility	Unknown (predicted high) (Bayley *et al.* 2010). Exclusively head and neck PGLs
*SDHB*	2001	Yes (2019)	AD	21% at age 50 and 42% at age 70 (Rijken *et al.* 2018)
*SDHC*	2000	Yes (2019)	AD	25% (non-probands)
*SDHD*	2000	Yes (2019)	AD. Maternally imprinted (paternal allele expressed)	54% at age 40, 50–68% at age 60 and 87% at age 70 (Andrews *et al.* 2018) (children of female carriers not included in analysis)
*SLC25A11*	2018	Yes (2021)	AD	Unknown
*TMEM127*	2010	Yes (2019)	AD	15% at age 40 and 32% at age 65 (Toledo *et al.* 2015)
*VHL*	1993	Yes (2019)	AD	50–84% (Castro-Teles *et al.* 2021)

The current list of PGVs known to be associated with PPGLs, their status on the current NHS panel, their inheritance pattern and their estimated penetrance where known. PGV, pathogenic germline variant.

As the pool of PGV carriers has increased, so too has the rate of tumour detection in these patients. Recent advances in nuclear medicine scanning techniques have contributed to this increase. Ebbehoj *et al.* describe an almost fivefold increase in the incidence of PPGLs detected in Denmark from 1977 to 2015 (Ebbehoj *et al.* 2021). With this increasing incidence, we can expect to encounter PGLs in ‘unusual’ anatomical locations with increasing frequency. PGLs of the mediastinum are thought to account for <2% of all PGLs (Gurrieri *et al.* 2018). No specific guidelines exist on their management. Here, we will perform a detailed literature review and discuss our experience with 18 such cases, highlighting the complexity in their variable behaviour and the challenges that exist in their management. As we encounter mediastinal PGLs with increasing frequency, we hope to improve our understanding of their behaviour as we develop a safe, evidence-based, personalised approach to their management.

All PPGLs have metastatic potential and are often associated with excess catecholamine production. Symptoms of catecholamine excess are non-specific and include headache, anxiety, diaphoresis, pallor and vomiting, among others. Symptoms often occur in episodes and can range from mild to life-threatening (catecholamine crises). Blood pressure is high during these episodes but can be normal otherwise. The episodic and non-specific nature of these signs and symptoms can make diagnosis difficult.

## Mediastinal PGLs

**Figure 2:**
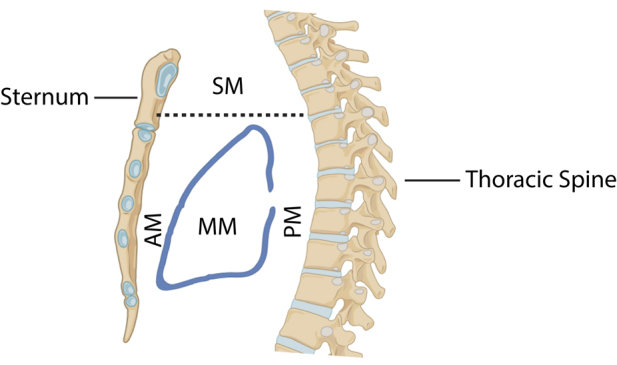
Anatomy of the mediastinum. SM, superior mediastinum. AM, anterior mediastinum. MM, middle mediastinum. PM, posterior mediastinum. Created in BioRender. Kemkem Y (2025). https://BioRender.com/o48k071.

Mediastinal PGLs are found in either the posterior mediastinum (originating from the paravertebral sympathetic chain) or in the anterior/middle mediastinum (originating from the intercarotid, subclavian, coronary, aorticopulmonary or paraaortic paraganglia). Cardiac PGLs are included in the latter and are most commonly found in the left atrium (Fig. 2).

The management of PPGLs generally involves surgical resection where possible. This is done to mitigate the risk of metastatic disease, avert any local pressure effects and/or manage catecholamine excess. Treatment options for metastatic PPGLs are limited, and the disease course is unpredictable, with 5-year survival rates varying from 12 to 84% (Hamidi *et al.* 2017). The most common sites of metastatic disease are the lymph nodes (80%), bone (71%), liver (50%) and lungs (50%) (Ayala-Ramirez *et al.* 2013). Pre-operative alpha blockade is essential in catecholamine-secreting tumours, and biopsies are best avoided due to the risk of precipitating a catecholamine crisis. Metastatic disease occurs in 15–25% of all PPGLs (Fishbein *et al.* 2021). It is increasingly apparent, however, that the pre-operative probability of developing metastatic disease varies according to several features, including tumour genotype, tumour size at diagnosis, the anatomical location of the primary tumour, its secretory phenotype and somatic changes within the tumour and its microenvironment (Ayala-Ramirez *et al.* 2011). A recent study utilised a machine learning model to estimate metastatic risk based on some of these features and demonstrated a sensitivity for the prediction of metastatic disease of 83% and a specificity of 92% (Pamporaki *et al.* 2023). As we move towards an era of personalised medicine in PPGLs, it is essential that we understand the clinical behaviour we can expect from these tumours across a spectrum of genotypes and phenotypes.

## Literature review

The literature on the management of mediastinal PGLs is limited to case reports and a small number of case series (Brown *et al.* 2008, Ghayee *et al.* 2009, Martucci *et al.* 2015, Gurrieri *et al.* 2018, Tella *et al.* 2020, Chan *et al.* 2022, Kanj *et al.* 2023; summarised in Table 2). Data on the genetic drivers associated with mediastinal PGLs are often lacking in these reports. Anterior/middle mediastinal PGLs pose a specific surgical challenge due to their proximity to major vessels and the heart. Most of the literature considers posterior mediastinal PGLs separate from middle/anterior mediastinal PGLs due to this aspect of their care (Table 2).

### Posterior mediastinal paragangliomas

Odze *et al.* published a case series and performed a review of the known cases of posterior mediastinal PGLs in 1990 and found evidence of increased catecholamine production when compared to anterior mediastinal PGLs. Within their series (*n* = 7), there was a high rate of metastatic disease. Their literature review found 48 cases with a 14.9% incidence of metastatic disease (Odze & Bégin 1990). Metastatic risk remains difficult to determine from our up-to-date review, but regarding the secretory phenotype, similar to Odze *et al.* we found multiple case reports of excess catecholamine production from posterior mediastinal PGLs (Kermenli & Azar 2019, Yue *et al.* 2019, Nam *et al.* 2020), including cases of biopsies and surgeries occurring without prior alpha blockade, resulting in critically elevated and often difficult-to-control intra-operative blood pressures (Muñoz-Largacha *et al.* 2017). Genetic testing was not carried out in these series.

### Anterior/middle mediastinal paragangliomas

Lamy *et al.* performed a similar review of anterior/middle PGLs in 1994 and found that in 79 cases there was a complete resection in 49%, a 26.6% incidence of metastatic disease, a 55.7% incidence of local recurrence and an operative mortality of 5.3% (Lamy *et al.* 1994). They found a 34.6% reduction in long-term survival in patients who had undergone a partial resection or biopsy compared to those who had a complete resection. While they highlighted the complexity of complete resection, they commented that ‘adequate exposure (of the tumour) is of the utmost importance’ and strongly recommended median sternotomy and cardiopulmonary bypass (CPB) if necessary to grant access to the relevant structures, to allow for mobilisation of the great vessels and to control bleeding if needed. A more recent series of 22 cases describes the use of CPB in 45.5% of their cohort (Gurrieri *et al.* 2018). Tumours that required CPB included tumours directly involved in cardiac structures and/or the distal ascending aorta and proximal aortic arch. Operative mortality was 4.5% in this series, 73% of the tumours were functional and metastatic disease was evident in 27.2% of patients at a median follow-up of 8.2 years. 95.4% of the tumours in this series were sympathetic in origin.

Brown *et al.* reported on 14 anterior/middle mediastinal PGLs in 2008. 85.7% had catecholamine excess confirmed pre-operatively. A genetic cause was diagnosed in 28.6% (one *SDHB*, one *SDHD* and two patients with Carney’s triad), but this is an underestimation, as at least 50% of the patients in this series had multiple PGLs and/or a positive family history of PPGLs. Tumours in this series ranged in size from 1 to 8 cm in maximum diameter (Brown *et al.* 2008).

Ghayee *et al.* published a similar series of ten mediastinal PGLs in the same year. In contrast, they found 100% of cases were due to an *SDHx* PGV (six *SDHB* and four *SDHD*), 70% had catecholamine excess and 60% eventually developed metastatic disease. Interestingly, they described cases of catecholamine excess (in three of four) and metastatic disease (in three of four) of their *SDHD* cohort. *SDHD*-related PGLs most commonly arise from the parasympathetic paraganglia of the head and neck, they rarely metastasise and are functional in <5% of cases (Baysal *et al.* 2000). In contrast, *SDHB* PGVs most commonly predispose to sympathetic, extra-adrenal or abdominal PGLs that are usually functional (Kantorovich *et al.* 2010). *SDHB*-related PPGLs carry the highest risk of metastatic disease (Andrews *et al.* 2018), which has been estimated to be 30% in some studies (Barbolosi *et al.* 2018). The mechanisms for this difference in risk are poorly understood. The cases in this series highlight the unpredictable nature of the disease that may develop in patients with *SDHx* PGVs. While the literature makes clear there is a ‘most common’ phenotype associated with each *SDHx* PGV, it is also true that a spectrum of disease exists, ranging from isolated, biochemically silent, non-aggressive tumours to multi-focal, functional, metastatic disease.

Regarding metastatic risk, much of the data that exists are drawn from retrospective reports from symptomatic patients managed in tertiary centres. A 2012 meta-analysis estimated the pooled incidence of metastatic disease in *SDHB* carriers (with and without appreciable disease) at 17% and in *SDHD* carriers at 8% (Van Hulsteijn *et al.* 2012). The generalisability of these findings is questionable. A detailed retrospective study of 872 patients with *SDHx* PGVs found a much lower risk of metastatic disease at 4.2% (95% CI 1.1–7.2%) by age 60 in non-proband *SDHB PGV* carriers (Andrews *et al.* 2018).

Considering PGLs from an anatomical perspective, the risk of metastatic disease also varies significantly. Ayala-Ramierz *et al.* reported the risk of metastatic disease to be highest with mediastinal PGLs (Ayala-Ramirez *et al.* 2011). In their 2011 paper, 69% of 13 mediastinal PGLs developed metastatic disease, 66% of 21 abdominal PGLs developed metastatic disease and 25.5% of 267 patients with phaeochromocytomas developed metastatic disease. Details on the genetic testing carried out in this cohort were incomplete.

### Cardiac PGLs

Cardiac PGLs have been reported in a number of case series and literature reviews (Martucci *et al.* 2015, Wang *et al.* 2015, Tella *et al.* 2020, Chan *et al.* 2022). These publications report a high rate of catecholamine excess (up to 93–96%, but this is not a consistent finding (Chan *et al.* 2022)). When genetic testing was carried out, there was also a high rate (at least 76.9% (Martucci *et al.* 2015)) of *SDHx*-related disease in cardiac PGLs. In one series of 15 cardiac PGLs, the tumour size ranged from 2.5 to 8 cm in maximum diameter (Martucci *et al.* 2015). Surgery generally involved CPB, and the data on metastatic disease are lacking. A number of these publications comment that the sensitivity of ^123^I-MIBG scanning was significantly reduced in relation to cardiac PGLs. In line with the latest European Association of Nuclear Medicine Guidelines, many authors now recommend ^68^Ga-DOTATATE PET CT as the chosen modality for PPGL diagnostic purposes (Taïeb *et al.* 2019) due to its improved sensitivity and specificity in identifying primary tumours as well as metastatic sites.

### Other thoracic PGLs and related conditions

Primary pulmonary paragangliomas that arise in chromaffin cells found within lung parenchyma are exceptionally rare and are not included in this series/review. There are also no cases of Carney-Stratakis syndrome (the dyad of familial PGL and gastrointestinal stromal tumour (GIST)) or Carney’s triad (PGL, GIST and pulmonary chondroma) included. These are rare conditions that share some clinical characteristics but have an entirely separate genetic basis. Carney-Stratakis syndrome arises in germline variants affecting *SDHB*, *SDHC* and *SDHD*, while Carney’s triad has a more uncertain genetic aetiology and has been described as a novel form of multiple endocrine neoplasia (Stratakis & Carney 2009). PGLs of Carney’s triad often arise in the mediastinum (Colwell *et al.* 2001). The presence of multiple thoracic lesions on imaging in these cases often causes confusion and requires careful interpretation and surgical planning.

## Cases

### Presenting complaint

Eighteen cases of mediastinal PGLs were identified via our neuroendocrine tumour multi-disciplinary meeting database. We used our electronic patient record to retrieve retrospective medical notes, radiological imaging and blood test results. The average age at presentation for patients with a mediastinal PGL was 46.8 years (range 19–80). 44.4% of cases were female. The mean follow-up time was 7.3 years (range 1–19). 33.3% of patients had biochemical evidence of catecholamine excess at initial presentation. A third of these (11.1% of cohort) had presented with symptoms of catecholamine excess. 33.3% were diagnosed incidentally on radiological imaging performed for an unrelated reason. 55.5% were diagnosed via radiological imaging performed as part of their routine *SDHx* screening. For 16.6%, *SDHx* carrier status was diagnosed via cascade screening, and this was their first screening scan. For 38.8%, there was a pre-existing diagnosis of an *SDHx* PGV (Table 3).

**Table 2:** A summary of the findings of the available literature on mediastinal paragangliomas. The rate of metastatic disease in these publications may include cases of metastasis from extra-mediastinal lesions and so may not relate to metastasis from a mediastinal primary (unless otherwise stated).

Publication	Type of report	Year of publication	Number of cases	Tumour location	Metastatic disease (%)*	PGV (%)	Catecholamine excess (%)
Lamy *et al.* (1994)	Literature review	1994	79	Anterior/middle	26.6%	Unknown	Unknown
Gurrieri *et al.* (2018)	Case series	2018	22	Anterior/middle	27.2%	Unknown	73%
Brown *et al.* (2008)	Case series	2008	14	Anterior/middle	Unknown	Unknown	92.3%
Ghayee *et al.* (2009)	Case series	2009	10	Anterior, middle and posterior	60%	100% (60% *SDHB* and 40% *SDHD*)	70%
Ayala-Ramirez *et al.* (2011)	Retrospective chart review	2011	13	Unknown	69%	Unknown	Unknown
Odze & Bégin (1990)	Case series	1990	7	Posterior	85.7%	Unknown	66.7% (of those tested)
Martucci *et al.* (2015)	Case series	2015	15	Cardiac	20%	76.9% (three *SDHB*, three SDHC and four *SDHD*)	93.3%
Chan *et al.* (2022)	Retrospective chart review	2022	22	Cardiac	5.3%	Unknown	31.5%
Kanj *et al.* (2023)	Retrospective chart review	2023	51	All	20% (6%^†^)	66% (46% *SDHB*, 17% *SDHD* and 3% *SDHC)*	44%
All combined^*^	-	-	233	All	39.2%	81%	67.3%
Quinn *et al.*	Case series	2025	18	All	22.2% (11.1%^†^)	72.2%	33.3%

* These means were calculated only from the series where full data were available.

^†^ Rate of metastatic disease that can be traced to mediastinal primary.

PGV, pathogenic germline variant.

**Table 3:** Details of our case series, including presenting complaint, diagnostic pathway (including biochemical and genetic analysis), pathological findings and incidence of metastatic disease in our cohort. PV, pathogenic variant.

Case	Presenting complaint	Age at diagnosis (years)	Tumour location	Tumour size	Functional imaging used	Functional imaging diagnostic	Catecholamine levels	Genetics- heterozygous germline pathogenic variant listed	SDHB IHC	Resected	Tissue at margin of resection	Disease evident on post-op scan	Metastatic disease	Duration of follow up
1	*SDHx* screening	19	Posterior mediastinum	1.5 × 1 cm	^131^I-MIBG scan	Yes	Normal range	*SDHB* c.587G>A p.(Cys196Tyr)	Negative	Yes	No	No	No	8 years
2	Incidental	33	Anterior	3 × 3 × 2 cm	^123^I-MIBG scan	Yes	Normal range	*SDHA* VUS* c.923C>T p.(Thr308Met)	Positive	Yes (partial)	Yes	Yes	No	19 years
3	*SDHx* screening	34	Anterior	0.6 × 0.6 cm	^68^Ga-DOTATATE PET CT	Yes	Normal range	*SDHB* c.689G>A p.(Arg230His)	NA	No – under surveillance	-	-	No	2 years
4	Incidental	62	Anterior	3.5 × 4 × 3.2 cm	^18^F-FDG PET	Yes	High	*SDHB* deletion of exon 1	Negative	Yes	No	No	No	7 years
					^123^I-MIBG scan	No								
5	*SDHx* screening	58	Anterior	1.5 cm	^123^I-MIBG scan	Yes	Normal	*SDHB* deletion of exon 1	Negative	Yes	Yes	No	No	6 years
6	*SDHx* screening	23	Posterior	1.6 × 1.6 cm	^123^I-MIBG scan	Yes	Normal	*SDHD* c.144_145dupCA p.(Ile49 fs)	Patchy	Yes	Yes	No	No	10 years
					Indium octreotide	No								
7	Incidental	49	Anterior	3.3 × 2.3 cm	^68^Ga-DOTATATE PET CT	Yes	High	None	NA	No – inoperable	-	-	Yes	2 years
8	*SDHx* screening	26	Anterior	0.4 × 0.7 cm	^68^Ga-DOTATATE PET CT	Yes	Normal range	*SDHD* c.242C>T p.(Pro81Leu)	NA	No – under surveillance	-	-	No	3 years
9	Incidental	48	Anterior	<1 cm	^18^F-FDG PET	No	Normal range	*SDHD* c.210G>C p.(Arg70Ser)	NA	No – under surveillance	-	-	No	1 year
					^68^Ga-DOTATATE PET CT	Yes								
10	Catecholamine excess	53	Middle	5 × 5 × 3 cm	^111^In-Octreotide	Yes	High	None	Unknown	Yes	Yes	Yes (not on all modalities)	No	11 years
11	*SDHx* screening	66	Middle	1.6 × 1 cm	^18^F-FDG PET	Yes	Normal range	*SDHD* c.144_145dupCA p.(Ile49 fs)	NA	No – under surveillance	-	-	No	6 years
					^123^I-MIBG scan	No								
					^68^Ga-DOTATATE PET CT	Yes								
12	*SDHx* screening	42	Anterior	0.6 × 0.6 cm	^68^Ga-DOTATATE PET CT	Yes	Normal range	*SDHD* c.169 + 5 G>A	NA	No – under surveillance	-	-	No	4 years
13	Incidental	67	Anterior	6.8 × 4.9 cm	^18^F-FDG PET	No	High (3-MTO only)	None	NA	No – under surveillance	-	-	No	6 years
					^123^I-MIBG scan	Yes								
					^68^Ga-DOTATATE PET CT	Yes								
14	*SDHx* screening	53	Cardiac	3.5 × 2.3 × 2.0 cm	^68^Ga-DOTATATE PET CT	Yes	Normal range	*SDHB* c.423 + 1G>A p.?	Negative	Yes	No	No	No	3 years
15	SDHx screening	40	Anterior/middle	1.0 × 0.8 cm	^68^Ga-DOTATATE PET CT	Yes	Normal range	*SDHD* c.242C>T p.(Pro81Leu)	NA	No – under surveillance	-	-	No	3 years
16	Incidental	80	Anterior	3.0 × .2.4 cm	^18^F-FDG PET	Yes	Normal range	None	Negative	No – inoperable	-	-	Yes	1.5 years
					^68^Ga-DOTATATE PET CT	Yes								
17	*SDHx* screening	47	Posterior	4.1 × 3.6 cm	^123^I-MIBG scan	Yes	High	*SDHB* c.268C>T p.(Arg90X)	Negative	Yes	Unknown	No	Yes	15 years
					^111^In-Octreotide	Yes								
18	Catecholamine excess	42	Unknown	Unknown	^123^I-MIBG scan	No	High	*SDHB* c.590C>G p.(Pro197Arg)	Unknown	Yes	Unknown	Unknown	Yes	14 years
					^68^Ga-DOTATATE PET CT	Yes								

* Genetic testing revealed a heterozygous sequence variant in exon 8 of the *SDHA* gene c. 923 > T p.(Thr308Met). At the time, this was not reported in the literature and remains a tepid variant of unknown significance (VUS). This variant has, however, been detected in a number of PPGL cases.

### Tumour location

**Figure 3:**
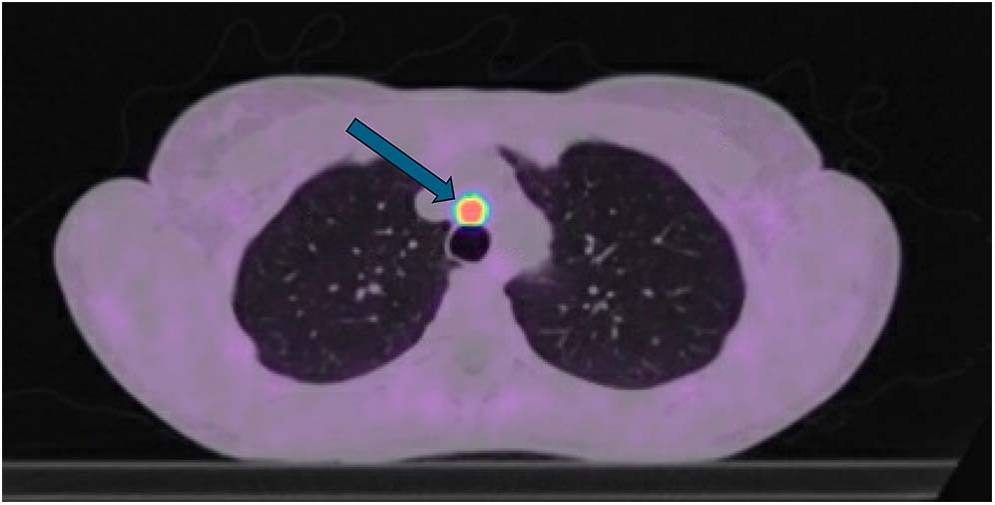
An image from a ^68^Ga-DOTATATE PET CT scan demonstrating a 6 mm anterior mediastinal PGL (arrowed). This lesion was not previously evident on MRI scanning.

72.2% of tumours were in the anterior/middle mediastinum, 16.6% were in the posterior mediastinum and 5.5% involved a cardiac PGL. Resection was attempted in 55.6% of cases. In those that did not undergo an operation (*n* = 7), four were <1 cm in diameter and have remained stable over a mean follow-up time of 4 years (range 1–6 years), two were in the context of metastatic disease (one in a patient with complicated cardiac anatomy and another in an elderly, frail man) and one had a major haemorrhage during mediastinoscopy for biopsy of a 6.8 × 4.9 cm PGL and opted for no further surgical intervention. Major haemorrhage from mediastinoscopy, biopsy and video-assisted thoracic surgery in this context have all been reported in the literature (Lamy *et al.* 1994). In our case, emergency sternotomy was required to control the bleeding. One further case (case 12) underwent mediastinoscopy for the removal of a 6 mm PGL, but histopathology revealed no tumour tissue in the resected sample and the area of avidity on ^68^Ga-DOTATATE PET CT has remained evident on subsequent scans but is stable in volume over 4 years (Fig. 3). The remaining surgeries were performed via median sternotomy or lateral thoracotomy.

### Genetics

All patients in our series have undergone genetic testing. 72.2% of this cohort carry an *SDHx* PGV (38.9% *SDHB* and 33.3% *SDHD)*. A further 5.6% carry a variant of uncertain significance (VUS) in *SDHA*. 22.2% of cases had no identifiable germline variant in any of the known PPGL-related PGVs. While this high rate of *SDHx*-related disease is consistent with much of the literature on mediastinal PGLs, there is a risk of bias towards a genetic basis in our cohort as 55% had a pre-existing diagnosis of an *SDHx* PGV. The diagnosis of a mediastinal PGL in these cases was made as a result of their inclusion in an appropriate screening programme.

**Figure 4:**
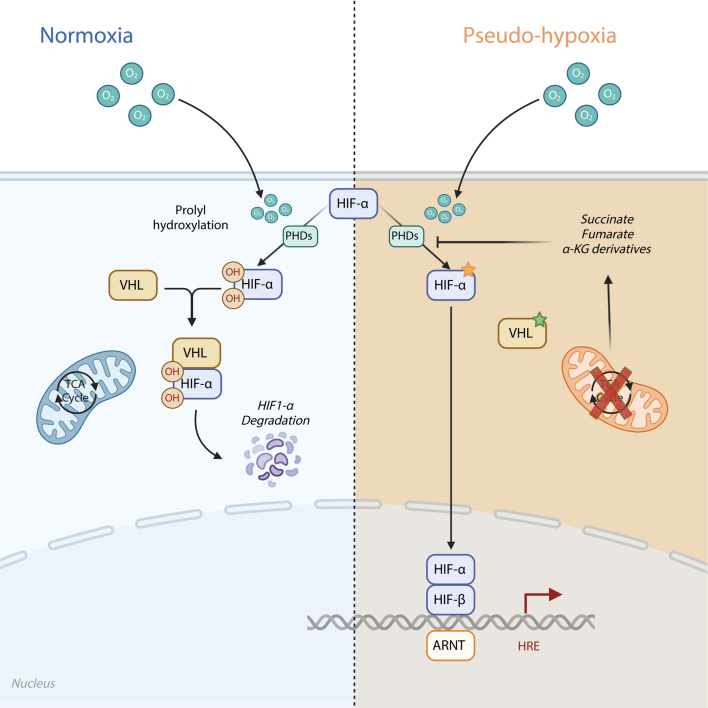
The hypoxia-inducible factor (HIF) pathway in normoxia (normal oxygen state) and in cluster 1 PPGL development. Created in BioRender. Kemkem Y (2025) https://BioRender.com/c89p666.

One of those cases not associated with a known PGV (case 7) occurred in the context of congenital cyanotic heart disease (CCHD), situs inversus, multiple corrective cardiac surgeries and chronic hypoxia (mean oxygen saturations of 82–86%). There is a known association between CCHD and the development of PPGLs (Opotowsky *et al.* 2015). This is likely mediated via chronic hypoxia in the same way pseudohypoxia contributes to the development of cluster 1 PPGLs (Fig. 4). PPGLs associated with CCHD generally present as PCCs, abdominal PGLs or head and neck PGLs. There are no reports of metastatic PPGLs in this context. To our knowledge, this is the first known case of a mediastinal PGL and the first known case of a metastatic PPGL (as discussed below – case 7) associated with CCHD.

### Metastatic disease

In total, 22.2% of our cohort have developed metastatic disease (50% of these had metastatic disease at diagnosis and 50% developed metastases at a median follow-up time of 8.5 years). Given the mutifocal nature of PPGL-related disease, it is not always possible to identify the source of metastatic disease. In one case of metastatic disease (case 7), the primary was a bladder PGL. In another (case 18), the patient had an abdominal and mediastinal PGL diagnosed and resected initially, followed by widespread metastatic disease presenting 7 years later. Corrected for these cases, the rate of metastatic disease in our cohort that can definitively be attributed to a primary mediastinal PGL was 11.1%. The metastatic rate from our literature review (when all series with complete data are combined) was 39.2%. In only one of these reviews is there clarity on the source of metastatic disease (Kanj *et al.* 2023) (where metastatic disease from a mediastinal primary occurred in 6% of their cohort over a median follow-up time of 8 years). Patients who developed metastatic disease in our cohort presented at an average age of 48 years. Those following a non-metastatic course presented at an average age of 44.6 years.

### Functional imaging

A variety of functional nuclear medicine scans were utilised in these cases, including: i) ^123^I/^131^I-labelled meta-iodobenzylguanidine (MIBG) scintigraphy/PET CT; ii) ^111^In-labelled octreotide scintigraphy; iii) ^18^F-labelled fluorodeoxyglucose (FDG) PET CT and iv) ^68^Ga-labelled DOTA-Tyr3-octreotate (DOTATATE) PET CT.

^123^I/^131^I-MIBG scintigraphy was used in 50% (*n* = 9) of cases. MIBG is structurally similar to noradrenaline and concentrates in catecholamine-secreting tissues. Many drugs interfere with MIBG uptake, including opiates, tricyclic antidepressants and many antihypertensive agents. The sensitivity of ^123^I/^131^I-MIBG SPECT in our series was 66.7%. Martucci *et al.* reported a sensitivity of 54.5% in 14 cardiac PGLs (Martucci *et al.* 2015). It has been reported that the sensitivity of ^123^I-MIBG scintigraphy is lowest in SDHB-related disease (Fonte *et al.* 2012). In our series, the false-negative results were in two SDHB cases and one SDHD case. It is also true that the sensitivity of ^123^I-MIBG scintigraphy is low in metastatic PPGLs as well as in head and neck PGLs (Timmers *et al.* 2009). For these reasons, this modality has largely been replaced for diagnostic purposes but does have a role in identifying patients who may be appropriate for ^131^I-MIBG therapy.

^111^In-Octreotide scintigraphy was used in three of our cases, with a sensitivity of 66.6%. ^111^In-Octreotide binds to somatostatin receptors (SSTRs) 2 and 5. Much of the literature on this technique reports improved sensitivity when compared to ^123^I-MIBG scintigraphy, especially in head and neck PGLs and mediastinal PGLs of parasympathetic origin. SPECT techniques have, however, largely been replaced by PET CT approaches.

^18^F-FDG PET CT was performed in five (four isolated and one metastatic) of our cases and had a sensitivity of 60%. ^18^F-FDG is taken into cells across glucose membrane transporters, where it accumulates in proportion to the cellular glycolytic rate. The sensitivity in PPGLs is reported to be 80–100% and performs best in SDHB-related disease (Taïeb *et al.* 2019). Regarding mediastinal PGLs, however, increased myocardial FDG uptake is thought to obscure small areas of avidity in the surrounding areas. This likely contributes to the reduced sensitivity of ^18^F-FDG PET CT seen in our series.

^68^Ga-DOTATATE binds to SSTR2 with the greatest affinity. Used together with PET CT, this technique has a sensitivity of 84–100% in diagnosing PPGLs (Gild *et al.* 2018). ^68^Ga has a half-life of 68 min, so the tracer must be synthesised on the day of the scan. This limits the availability of this scan to certain tertiary centres. In our series, ^68^Ga-DOTATATE PET CT was used in 61.1% of cases (*n* = 11) and had a sensitivity of 100%.

The recommendations from the latest international consensus guidelines on initial screening and follow-up of asymptomatic *SDHx* carriers somewhat reflect our findings. They state PET CT is the preferred modality of functional imaging over SPECT CT approaches due to technical advantages, including a shorter imaging procedure. While there is no consensus on the modality of PET CT used, there is reference to the EANM-SNMMI joint guidelines that state ^68^Ga-DOTATATE should be first line, with ^18^F-FDG PET CT used in cases where ^68^Ga-DOTATATE is not available (Amar *et al.* 2021).

## Treatment options

While surgery with complete resection is the first-line treatment option in mediastinal PGLs, this approach is not always possible. The vascular and often functional nature of these tumours, as well as their proximity to great vessels and major structures, makes surgery challenging. With the increased sensitivity of nuclear medicine scans, we are also detecting an increased number of small mediastinal PGLs which may not be detected on alternative imaging modalities. There is no clarity on the metastatic risk associated with these small tumours. We report six cases in our series that measured <1.6 cm in maximum diameter on imaging but had clear increased avidity on nuclear medicine scans (all detected on ^68^Ga-DOTATATE PET CT). All six occurred in the presence of an *SDHD* PGV and were associated with normal catecholamine levels. Five out of six cases have remained stable, with no visible growth on imaging throughout an average follow-up period of 4 years (range 1–6 years). One cardiac PGL has increased in size from 0.4 × 0.7 to 2 cm in diameter over 4 years but has remained biochemically silent. Kanj *et al.* report a median growth of 0.2 cm per year in 13 mediastinal PGLs that did not undergo surgical resection (Kanj *et al.* 2023).

In our series ten patients underwent surgical resection. In one case (case 2), there was evidence of tumour tissue at the margin of excision on pathological assessment, as well as appreciable disease on post-operative ^123^I-MIBG scintigraphy. This patient underwent ^131^I-MIBG therapy, which was well tolerated. Over the following 19 years, serial ^123^I-MIBG scintigraphy, CT and MRI scans have demonstrated unchanged radiological features of the lesion of interest, with no distant metastases and unremarkable serum and urinary catecholamine levels. An additional three patients had tumour tissue at the margin of excision, but no disease was evident on post-operative imaging. There has been no evolution of disease on imaging in these cases over follow-up ranging from 6 to 11 years. Of the remaining six patients who underwent surgical resection, four had complete resection and have remained disease-free over follow-up ranging from 3 to 10 years. For the remaining two patients, information on tumour tissue at the margin of excision is unavailable. Both developed metastatic disease as described below (cases 17 and 18).

## Metastatic disease

Treatment of all PPGLs should be dictated by an expert multidisciplinary team. This is especially true in cases of metastatic disease where the evidence base is limited. Surgical debulking of primary tumours in metastatic disease is controversial where one large study suggested no benefit (Ellis *et al.* 2013), while some more recent studies suggested improved overall survival with this approach (Roman-Gonzalez *et al.* 2018). Alternative treatment strategies include radionuclide therapy (including ^131^I-MIBG, ^90^Y-DOTATATE or ^177^Lu-DOTATATE therapies), chemotherapy (cyclophosphamide, vincristine and dacarbazine or temozolomide), or tyrosine kinase inhibitors. Many of these approaches are currently undergoing clinical trials to determine long-term safety, efficacy and effect on overall survival.

Two of our cases were diagnosed with metastatic disease at the time of their diagnosis (cases 7 and 16). Case 7 had multifocal disease with a bladder PGL and two positive lymph nodes previously resected. The 3 cm mass adjacent to the left atrium was deemed inoperable due to complex cardiac anatomy (background of CCHD as previously discussed). The patient was started on alpha-blockade with doxazosin and the ‘cold’ somatostatin analogue (SSA) lanreotide. SSAs are currently licensed for use in gastroenteropancreatic neuroendocrine tumours (GEP NETS) and in carcinoid tumours. There is good evidence that SSAs have effective anti-secretory and anti-proliferative effects in GEP NETs (Rinke *et al.* 2009), but evidence for their use in PPGLs is limited to case reports and case series (Van Hulsteijn *et al.* 2013). Lanreotide exerts its inhibitory effect via SSTR 2 and 5. PPGLs are known to have variable SSTR expression; however, Fischer *et al.* have recently reported that SSTR 2 expression is associated with metastatic PPGLs as well as *SDHx*-related PPGLs (Fischer *et al.* 2023). In this study, six patients with metastatic PPGLs had control of disease with SSA therapy. An exploratory phase II study of lanreotide in metastatic PPGLs (LAMPARA) is currently underway. In our case, normetadrenaline levels reduced from 7073 to 4458 pmol/L after initiation of SSA therapy, with slightly reduced tumour volume over 27 months.

Case 16 had a 3.4 cm anterior mediastinal PGL diagnosed with thoracotomy performed to assess the feasibility of resection. The tumour was deemed inoperable, and a visible lymph node was biopsied, which was consistent with a metastatic PGL deposit. *SDHB* IHC was negative on this sample, but genetic testing has not identified a PGV. Following NET MDT review, the patient underwent three cycles of ^177^Lu-DOTATATE peptide receptor radionuclide therapy (PRRT). This was tolerated well and there has been no progression of disease on ^68^Ga-DOTATATE PET CT over 18 months.

PRRT is similar to cold SSA therapy but produces its antitumour effect by delivering a dose of radiation to neuroendocrine tumour cells. It is licensed for use in GEP NETS but there is an increasing body of evidence demonstrating its safety and efficacy in metastatic PPGLs (Severi *et al.* 2021). ^131^I-MIBG therapy is currently the only approved treatment strategy for metastatic PPGLs (approved by the European Medicines Agency and the U.S. Food and Drug Administration). It has been shown to be a safe and efficacious treatment strategy in this context (Pryma *et al.* 2019). Some reports have suggested that PRRT performs better in terms of progression-free survival in some scenarios (Prado-Wohlwend *et al.* 2022), but in cases of MIBG avidity, ^131^I-MIBG therapy continues to have a role.

Two further patients (cases 17 and 18) were diagnosed with distant metastatic disease at 10 and 7 years after their initial presentation.

Case 17 developed metastatic disease 10 years after the initial mediastinal tumour resection. Imaging, biochemical and pathological findings were reassuring at the time of surgery. Ten years post-operatively, the patient presented with a pathological neck of femur fracture and was found to have raised catecholamines and widespread metastatic disease. There was a large tumour at the primary operative site consistent with recurrence. A complex treatment course followed that involved ^131^I-MIBG therapy, PRRT (four cycles of 7.4 GBq ^177^Lu-DOTATATE therapy), surgical resection of spinal lesions and repeated courses of radiotherapy at T9 and T10 due to impending spinal cord compression. Care was further complicated by osteonecrosis of the jaw and a simultaneous diagnosis of breast cancer. The patient died 15 years after the initial diagnosis and 5 years after the diagnosis of metastatic disease.

Case 18 had abdominal and mediastinal PGLs removed initially. Metastatic disease presented 7 years later with raised metanephrines and evidence of liver and retroperitoneal disease on MRI and ^68^Ga-DOTATATE PET CT. These lesions were not avid on ^123^I-MIBG scintigraphy. The patient underwent four cycles of 7.4 GBq ^177^Lu-DOTATATE therapy. Disease remained stable on imaging over 2 years. At 3 years post-PRRT, MRI and ^68^Ga-DOTATATE PET CT demonstrated similar disease distribution but an increase in the volume and avidity of some lesions. Other than some pain at the site of a rib metastasis, there are no specific symptoms reported.

While many of the treatment strategies employed in these cases are not licensed for the treatment of metastatic PPGLs, there is a strong evidence base for their use. As we work towards a personalised approach to the management of PPGLs, there will be further treatments that are likely to be effective in the management of these patients. Our unique understanding of the pathophysiology of PPGLs has highlighted a number of specific treatment targets, including (but not limited to) the mTOR signalling pathway (with the mTOR inhibitor everolimus) and the transcription factor hypoxia-inducible factor 2α (with the HIF-2α inhibitor belzutifan). The latter is currently under clinical trial as a monotherapy for the treatment of advanced PPGLs, with an estimated completion date of 2027.

## Conclusion

Here, we report our experience with 18 mediastinal PGLs. The strengths of this series include the complete data for genetic testing, metastatic disease and rates of catecholamine secretion. We have also discussed the stability of five small anterior/middle mediastinal PGLs (<1.6 cm in diameter) over an average follow-up of 4 years (range 1–6 years), which has not been published before. One cardiac PGL increased in size from 0.4 × 0.7 to 2 × 2 cm over 4 years. All six were diagnosed on ^68^Ga-DOTATATE PET CT, none have demonstrated excess catecholamine secretion and they have all arisen in the presence of an *SDHD* PGV. The increase in detection of these small lesions is due to increasing patient numbers and the increased sensitivity of ^68^Ga-DOTATATE PET CT. In the absence of catecholamine secretion and local compressive effects, their clinical relevance is unclear, and early surgical intervention may not be beneficial. An MDT approach is essential to ensure any intervention is carefully planned.

We report a metastatic rate (that can be traced to a primary mediastinal lesion) of 11.1% and a catecholamine excess rate of 33.3% in this series. Isolated mediastinal PGLs that were associated with catecholamine secretion (*n* = 3) tended to be larger, with tumour dimensions ranging from 3.5 × 4 to 6.8 × 4.9 cm. Regarding the risk of metastatic disease, we report a much lower rate than previous case series (estimated to be 39.2% – summarised in Table 2). This difference can likely be explained by the inclusion, in our cohort, of smaller lesions detected by ^68^Ga-DOTATATE PET CT. Previous case series utilised diagnostic tools that may only demonstrate sensitivity in diagnosing larger mediastinal PGLs, which are more likely to be more advanced and thus have a higher risk of metastasis. For example, high FDG uptake in the myocardium is known to obscure adjacent lesions and so make it difficult to diagnose small mediastinal PGLs with ^18^F-FDG PET CT. The sensitivity of ^123^I/^131^I-MIBG scintigraphy in the diagnosis of cardiac PGLs is also known to be reduced (Martucci *et al.* 2015). The increase in the use of highly sensitive diagnostic tools in PGL detection has created a new cohort of patients. Management strategies should be developed with this in mind.

We have also discussed the utility of cold SSA therapy and PRRT/MIBG therapy in the management of inoperable and/or metastatic disease. Many alternative therapies for metastatic PPGLs are currently under trial and are likely to yield a more personalised approach to the management of this condition.

The main weakness of this series is the retrospective nature of much of the data collection. Our rates of metastatic disease are also based on variable follow-up times. Given the significant latency in the development of metastatic disease in many PPGL cases, it is possible the incidence we report of metastatic disease is an underestimation.

Mediastinal PGLs most commonly arise in the context of a PGV in *SDHx*. ^68^Ga-DOTATATE PET CT is the most sensitive imaging modality in our cohort and can lead to the identification of small neuroendocrine tumours. In a time of personalised care, multiprofessional decision-making guides conservative, surgical and non-surgical treatment approaches, with the majority of patients having a good outcome.

## Declaration of interest

Mark Quinn is an Early Career Editor of *Endocrine-Related Cancer*. Mark Quinn was not involved in the review or editorial process for this paper, on which he is listed as an author. We declare that there is no conflict of interest that could be perceived as prejudicing the impartiality of this work.

## Funding

This work did not receive any specific grant from any funding agency in the public, commercial or not-for-profit sector.
